# Effect of different polishing techniques on surface roughness and bacterial adhesion of three glass ionomer-based restorative materials: *In vitro* study

**DOI:** 10.4317/jced.56616

**Published:** 2020-07-01

**Authors:** Hoda S. Ismail, Ashraf I. Ali, Mohammed A. Abo El-Ella, Salah H. Mahmoud

**Affiliations:** 1 Assistant Lecturer, Operative Dentistry Dept, Faculty of Dentistry, Mansoura University, Egypt; 2 Associate Professor, Operative Dentistry Dept, Faculty of Dentistry, Mansoura University, Egypt; 3 Professor, Microbiology and Immunology, Faculty of Medicine, Mansoura University, Egypt; 4 Clinical Professor and Chairman of Operative Dentistry, Faculty of Dentistry, Mansoura University, Egypt

## Abstract

**Background:**

Although many reports concluded that polishing of glass ionomers is crucial for smoother surface and limiting the adhesion of cariogenic bacteria, there is no specific surface treatment protocol recommended. A novel material in the same category was released recently claimed to have surface smoothness comparable to resin composite and bacterial adhesion less than other types of glass ionomers. In this study, different polishing systems were tested with three glass ionomers one of them is the novel material to find the most appropriate polishing protocol. Objectives: To evaluate and compare the surface roughness and bacterial adhesion to resin modified glass ionomer, bioactive ionic resin and conventional glass ionomer restorative materials after different polishing protocols in vitro.

**Material and Methods:**

The materials tested includes resin modified glass ionomer, bioactive ionic resin, and conventional glass ionomer. The polishing protocols were divided into four groups: group 1 = (Mylar matrix strips, Control), group 2 = (one-step, PoGo), group 3 = (two-step, Prisma Gloss) and group 4 = (three-step, Sof-Lex). From each material, eleven cylindrical specimens were prepared for each group according to the manufacturers’ instructions. The surface roughness for all specimens was measured using atomic force microscope in tapping mode. the same specimens were subjected to bacterial adhesion testing after being coated with artificial saliva. Data were analyzed with two-way analysis of variance followed by Post hoc multiple comparisons.

**Results:**

The highest Ra and *S. mutans* adhesion values were recorded for all materials in two-step group. The lowest Ra and *S. mutans* adhesion values were seen in one-step and three step groups.

**Conclusions:**

One-step polishing system was more effective and may be preferable for polishing of the three studied glass ionomer-based materials compared to two-step and three-step systems.

** Key words:**Activa bioactive restorative, glass ionomer, surface roughness, bacterial adhesion, surface treatment.

## Introduction

Colonization of cariogenic bacteria on margins of restorations can contribute to increase in the incidence of secondary caries, which is one of the major reasons for replacement of restorations (1). So, efforts have been made to minimize or prevent plaque formation on restorative materials (2). Numerous *in vitro* and *in vivo* models have investigated both the adhesion of various microorganisms to dental restorations and the mechanisms involved therein (3,4).

Glass ionomer restorative materials (GIs) offer reasonable esthetics, chemical bonding, fluoride release and caries inhibiting potentials without extensive sound tooth structure preparation (5). Many types and modifications have been developed to enhance the mechanical and esthetic properties of the material (6). Resin modified glass ionomer cements (RMGI) are one of these modifications where a second resin polymerization reaction is supplemented to the fundamental acid-base reaction takes place (7). Recently, a new bioactive resin materials was released to dental market (ACTIVA, Pulpodent, USA). The manufacturer claim that it delivers all the advantages of glass ionomers in a strong, resilient, resin matrix that will not chip or crumble (8).

Surface roughness influences the stain resistance and bacterial adhesion of the restoration (9), so several techniques for finishing and polishing were assessed. In recent years, investigators have tried to use one-step polishing systems to achieve a high quality surface. Studies (10,11) have shown that one-step techniques are superior or at least comparable to multi-step techniques however, in some cases, the results were product related (12).

Unfortunately there is usually a problem during the finishing and polishing of GIs due to the heterogeneity of the composition and the difference in hardness between the inorganic fillers and the matrix that leads to non-uniform abrasion (13). It is still unclear whether the use of one-step, two-step or three-step polishing techniques substantially affects the surface behavior of GI-based restorative materials.

The aim of this study was to evaluate the surface roughness and *S. mutans* adhesion on the surface of three different glass ionomer-based restoratives subjected to different polishing techniques. The null hypotheses tested were that neither the type of restorative material nor the type of polishing system would provide distinguishable textural features; *S. mutans* adhesion to restorative materials would not depend on the surface roughness generated after different polishing systems.

## Material and Methods

I. Specimen Preparation

The materials tested in the current study includes resin modified glass ionomer (Fuji II LC) (RMGI), bioactive ionic resin (ACTIVA) (BIR), and conventional glass ionomer (EQUIA Fil) (CGI). A total of 132 specimens (44 specimens of each restorative material) were prepared in a specially designed mold (10 mm diameter × 2 mm depth). The specimens were prepared by a standardized method in which the restorative material was pressed into the mold between two glass slides covered by Mylar strips (SS White, USA). All specimens were prepared, mixed and dispensed according to the manufacturer’s instructions.

The surface of the resin modified glass ionomer (RMGI) and bioactive ionic resin (BIR) specimens was cured for 40 seconds by a curing device (Elipar Deep Cure, 3M ESPE, St. Paul, MN, USA) operating at 1000 mW/cm2. For the CGI specimens, G-Coat Plus was applied on both surfaces using a micro-brush and photo cured for 20 seconds. All specimens were stored in distilled water at 37°C for 24 hours in an incubator (BTC, Model: BT1020, Egypt) prior to the finishing and polishing procedures.

II. Polishing Treatment

From each material, four groups were randomly created (n=44).

Group 1: These specimens were kept without finishing and polishing after removal of the Mylar strips and served as a control group 

Afterwards, the surfaces of the remaining specimens were treated with a super-fine grit finishing diamond (25 μm) attached to a high speed hand piece to simulate the initial contouring of restorative materials. A low-speed hand piece (STRONG, Model: STRONG 204, Korea) (10,000 rpm) with uniform pressure and planar motion was used for all polishing procedures according to the manufacturer’s instructions.

**Figure 1:**
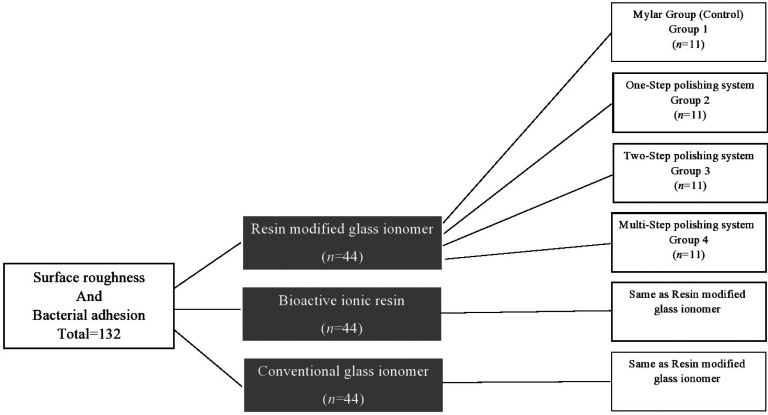
Flow chart of the study design.

Group 2: The specimens were polished with a Pogo diamond micro polisher disc for 40 seconds. Group 3: The specimens were polished using an Enhance Foam polishing cup with Prisma Gloss fine and super fine pastes for 30 seconds. Group 4: The specimens were polished with a Sof-Lex aluminum oxide disc system at descending sequence of abrasiveness; medium, fine and superfine. The detailed study design is shown in Figure 1.

III. Surface Roughness Test

The surface roughness test was performed with the Digital Instruments Thermo Microscope AFM (Autoprobcp, Santa Barbara, CA, USA) operating in tapping mode. Tapping mode AFM uses an oscillating tip (typically at a frequency of 320 kHz) at an amplitude of approximately several tens of nm when the tip is not in contact with the surface. When the oscillating tip moves towards the surface, it begins to touch the surface causing the amplitude to be reduced. This reduction is used to measure the surface topographic features (14). AFM images were analyzed using the Park Scientific Instruments software package supplied with the AFM instrument, and the surface roughness of each specimen was evaluated as the average roughness height (Ra) of the AFM topographical images.

VI. Bacterial Adhesion Test 

The same specimens used for surface roughness test were used in bacterial adhesion test after packing of every specimen in a dry plastic sterile bag and autoclaving at 121°C.

1. Saliva coating 

Artificial saliva was prepared as was described by Rosentritt *et al.* (15) at Faculty of Pharmacy, Mansoura University, then stored at −20 °C before the test. The specimens were treated with 90% vol. ethanol for 10 min to remove traces of lipids and proteins from the surfaces. After that, they were transferred into culture plates that were filled with 1.5ml artificial saliva and incubated for 1 hour at 37 °C.

2. Suspension preparation and adhesion steps 

A reference strain of *S. mutans* (ATCC 25175) was used for this test. The bacterial suspension was prepared with a concentration of 0.5 McFarland.

The adhesion test was performed on 24-well tissue culture plates. Each material disc was placed at the bottom of a well, which was then filled with two ml of fresh broth and 20 µl of cell suspension. These plates were incubated at 37 °C for 4 hours. Then, the tested discs were washed three times with 5 ml of a sterile 0.9% NaCl solution to remove non-adhering cells. Following this step, each disc was placed in a glass tube containing 1 ml of saline solution and placed in an ultrasonic bath operating at 47 kHz and 234 W for 6 minutes to detach bacteria adhering to the disc surfaces. Ten milliliters of fresh broth were added to each tube after removal of the disc. Ten microliters of the final solution from each tube were cultured on blood agar, and all culture plates were then incubated at 37 °C for 24 hours. The colonies were counted at the end of the incubation period with a magnifying lens, and the number of colony-forming units per milliliter of suspension (CFUx103/mL) were calculated. Statistical analysis was performed using SPSS® software (SPSS® Statistics 20.0.0; SPSS Inc., Chicago, IL, USA). Two-way ANOVA, Tukey’s honest significant difference (HSD) post hoc test and Pearson’s correlation coefficient (r) test were used. A result was considered to be statistically significant if *p*<0.05.

## Results

**Figure 2:**
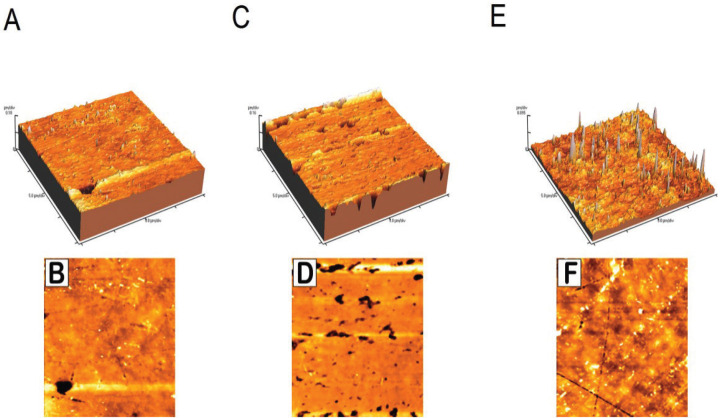
Atomic force microscope (AFM) images used for estimating the surface roughness parameter (Ra). A, C and E are the three dimensional images of 3 control samples of RMGI, BIR and CGI respectively; B, D and F are the two dimensional images of the corresponding samples.

The results of two-way ANOVA revealed that ‘type of restorative material’ and ‘polishing technique’ significantly affected mean Ra values (*p*<0.05), the interaction of both variables were also significant (*p*<0.05). The mean Ra values and standard deviations for all groups are presented in Table 1. Tukey’s HSD multiple comparisons showed that the highest mean surface roughness values were obtained using CGI followed by BIR and RMGI (Fig. 2). There were significant differences among the three groups. In terms of polishing techniques, the highest surface roughness values were obtained for the two-step group. The lowest surface roughness values were recorded when using a Mylar matrix followed by the one-step and three-step groups (Table 1).

**Table 1:**
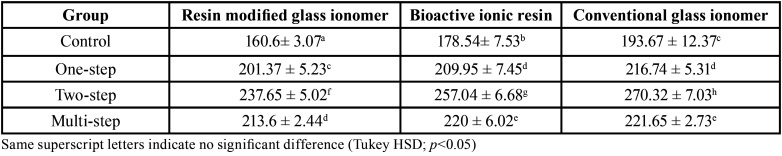
Mean ± Standard Deviation of Surface Roughness Values (nm).

**Figure 3:**
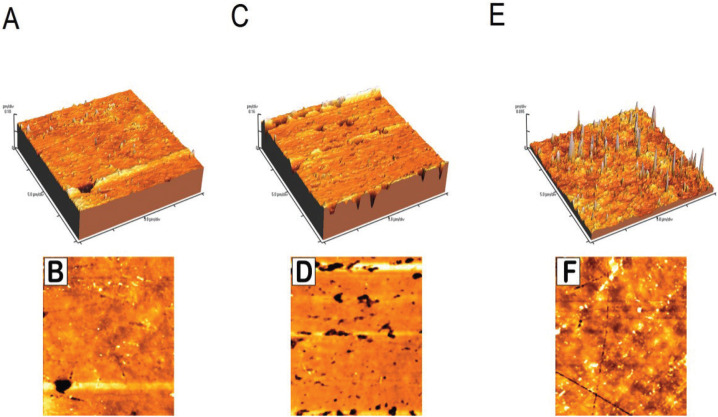
*S. mutans* growth on blood agar culture. A, B and C represent S. mutans colony counts of 3 control samples of RMGI, BIR and CGI respectively; black arrows point at *S. mutans* colonies.

The results of two-way ANOVA showed that the values of bacterial adhesion means differed significantly among the restorative materials and polishing systems, the interaction of both variables was also significant (*p*<0.05). The means and standard deviations of the number of colonies (CFUx103/ml) for all groups are presented in Table 2.Tukey’s HSD showed that the adhesion values were the highest for CGI followed by those for BIR and RMGI (Fig. 3). In terms of the polishing techniques, the amount of bacterial adhesion were the lowest on the surface of Mylar strip and one-step groups, while the two-step group produced the highest bacterial adhesion (except for BIR group) (Table 2). Pearson correlation showed significant positive relation between surface roughness and bacterial adhesion values for all materials and polishing systems used in the study (r = 0.623, *p* = .05).

**Table 2:**
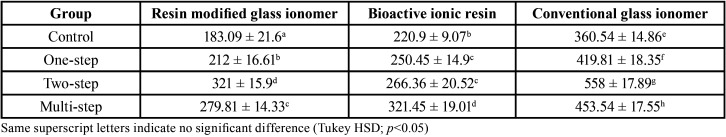
Mean ± Standard Deviation of Bacterial Adhesion values (CFUx103/ml).

## Discussion

In accordance with the literature, the smoothest surfaces were produced by curing the materials against Mylar matrix strips. Unfortunately, this procedure is often clinically insufficient because post curing finishing procedures have to be performed to remove excess material, obtain the correct anatomical form and polish the surfaces (16). The AFM was used in tapping mode not to affect the specimens’ surfaces, thus allowing other tests to be performed on the same specimens and correlations to be made between these tests (14).

Based on the results, the null hypothesis that textural features of glass ionomer-based restorative materials would not affected by polishing technique was rejected. The outcome of the roughness test of the current laboratory study and other studies (12,17) revealed that the smoothest restorative material surfaces were obtained with Mylar strips in all control groups of the three materials.

In the present study, RMGI and BIR exhibited lower surface roughness than did CGI. This may be attributed to the smaller particle size of the two materials than that of CGI (Fuji II LC: 4.5 μm, ACTIVA: 4 μm, EQUIA: 7 μm) (18). Additionally, the presence of resin in the composition of RMGI and BIR may help remove fine chips from the surface of the material during polishing, thereby producing a smooth surface. Although the particle sizes of both RMGI and BIR were nearly the same, the results showed that the RMGI and BIR materials did not represent a homogeneous group with respect to surface roughness compared to CGI. This phenomenon can be attributed to other parameters that may contribute to a material’s surface finish such as differences in shape, distribution, number of particles and type of resinous matrix and ultimate degree of curing reached (19).

Regarding the polishing techniques used in the current study, one-step group revealed the smoothest surfaces for all groups but significantly rougher than those created with Mylar matrix (control group). This may be attributed in part to the use of fine diamond powders that are harder than aluminum oxide. The specimens polished with three-step system revealed better surface texture compared to two-step group specimens; this result could be explained by the decreasing order of abrasiveness of the polishing discs used, which enhanced the final surface texture. The two-step system rough surfaces may be attributed to the porous synthetic foam pad and polishing pastes which preferentially abraded the soft polysalt and resin matrix at a high rate and had a minimal effect on the filler particles of these materials that were left protruding from the surfaces.

The Streptococcus *S. mutans* was selected for this study as it is regarded as a major participant in dental caries (20). The adhered cells were removed for quantification after four hours because biofilm formation in the oral cavity is normally completed in 2-4 hours (21).

Based on the results of the current study, the null hypothesis that *S. mutans* adhesion to restorative materials would not depend on the surface roughness generated after using different polishing systems was rejected. In the current study, the lowest viable *S. mutans* counts occurred on RMGI specimens followed by BIR and CGI irrespective of surface treatment, this result can be attributed to the surface roughness values of the three materials. Surface roughness is considered one of the most important characteristics of restorative materials implicated in the bio adhesion process (22).

In this study BIR group showed higher adhesion value when finished with three-step than two-step systems contradictory to their roughness values. This can be attributed to other possible factors affecting bacterial adhesion other than surface roughness.

The chemical composition of the surface is important for bacterial adhesion. It was reported that low surface free energy (SFE) bacteria (like *S. mutans*) adhere preferentially to low SFE surfaces, more plaque is formed on hydrophobic materials like resins (23). This may also, in addition to surface roughness values, explain why BIR attracted more bacteria on its surface than RMGI as it contains more organic content. Conventional glass ionomer was the material had the greatest bacterial adherence with respect to that of the other restorative materials, away from its high Ra values, the bacterial exposure time was short, and so, no observed adverse effects on bacteria were derived from fluoride release.

The current study reported a significant positive correlation between surface roughness and bacterial adhesion. Despite the comparison methods found in the literature, the correlation between surface roughness and bacterial adhesion related to this study was also found by other investigators (24,25). However, some studies have not found such correlation (26,27).

Clinical validity of the different surface treatment systems of glass ionomer–based restorative materials, especially bioactive ionic resin material as it recently released to the dental market, should be evaluated.

## Conclusions

Within the research limitations, the findings of the present study revealed that the surface roughness and bacterial adhesion of glass ionomer-based restorative materials were significantly affected by polishing protocols. Considering the reduced number of steps, the current one-step polishing system appears to be more effective than the two-step and multi-step systems and may be preferable for polishing of the current materials. Regardless of the polishing techniques, surface roughness and bacterial adhesion are material dependent. Resin modified glass ionomer has the smoothest surface and the least bacterial adhesion than other tested materials after all polishing protocols.
